# Optimization of intraperitoneal aerosolized drug delivery using computational fluid dynamics (CFD) modeling

**DOI:** 10.1038/s41598-022-10369-8

**Published:** 2022-04-15

**Authors:** Mohammad Rahimi-Gorji, Charlotte Debbaut, Ghader Ghorbaniasl, Sarah Cosyns, Wouter Willaert, Wim Ceelen

**Affiliations:** 1 Department of Human Structure and Repair, Faculty of Medicine and Health Sciences, Ghent University, Corneel Heymanslaan 10, route 1275, 9000 Ghent, Belgium; 2 IBiTech - Biommeda, Faculty of Engineering and Architecture, Ghent University, Corneel Heymanslaan 10, 9000 Ghent, Belgium; 3 Department of Mechanical Engineering, Faculty of Engineering, Vrije Universiteit Brussel (VUB), Pleinlaan 2, 1050 Brussels, Belgium; 4 Cancer Research Institute Ghent (CRIG), Ghent, Belgium

**Keywords:** Computational biophysics, Cancer, Surgical oncology

## Abstract

Intraperitoneal (IP) aerosolized anticancer drug delivery was recently introduced in the treatment of patients with peritoneal metastases. However, little is known on the effect of treatment parameters on the spatial distribution of the aerosol droplets in the peritoneal cavity. Here, computational fluid dynamics (CFD) modeling was used in conjunction with experimental validation in order to investigate the effect of droplet size, liquid flow rate and viscosity, and the addition of an electrostatic field on the homogeneity of IP aerosol. We found that spatial distribution is optimal with small droplet sizes (1–5 µm). Using the current clinically used technology (droplet size of 30 µm), the optimal spatial distribution of aerosol is obtained with a liquid flow rate of 0.6 mL s^−1^. Compared to saline, nebulization of higher viscosity liquids results in less homogeneous aerosol distribution. The addition of electrostatic precipitation significantly improves homogeneity of aerosol distribution, but no further improvement is obtained with voltages higher than 6.5 kV. The results of the current study will allow to choose treatment parameters and settings in order to optimize spatial distribution of IP aerosolized drug, with a potential to enhance its anticancer effect.

## Introduction

Peritoneal metastases (PM) are a common manifestation of gastro-intestinal and gynecological cancers. Compared to other metastatic locations such as the liver, systemic chemotherapy is less active against PM, with a survival typically less than 10 months^1^. In addition, the quality of life of these patients is often poor due to debilitating symptoms such as obstruction or ascites formation^2–4^. Over the past decades, the role of intraperitoneal drug delivery (IPDD) has been explored as an addition to systemic chemotherapy^5^. During IPDD, PM are directly exposed to chemotherapy, while systemic toxic effects remain limited due to the pharmacological advantage conferred by the peritoneum-plasma barrier.

Different methods of IPDD are currently used, including catheter-based repeated intraperitoneal (IP) instillation and hyperthermic intraperitoneal chemoperfusion (HIPEC) in association with cytoreductive surgery (CRS)^6,7^. However, many patients present with widespread and/or unresectable disease.

**Figure 1:**
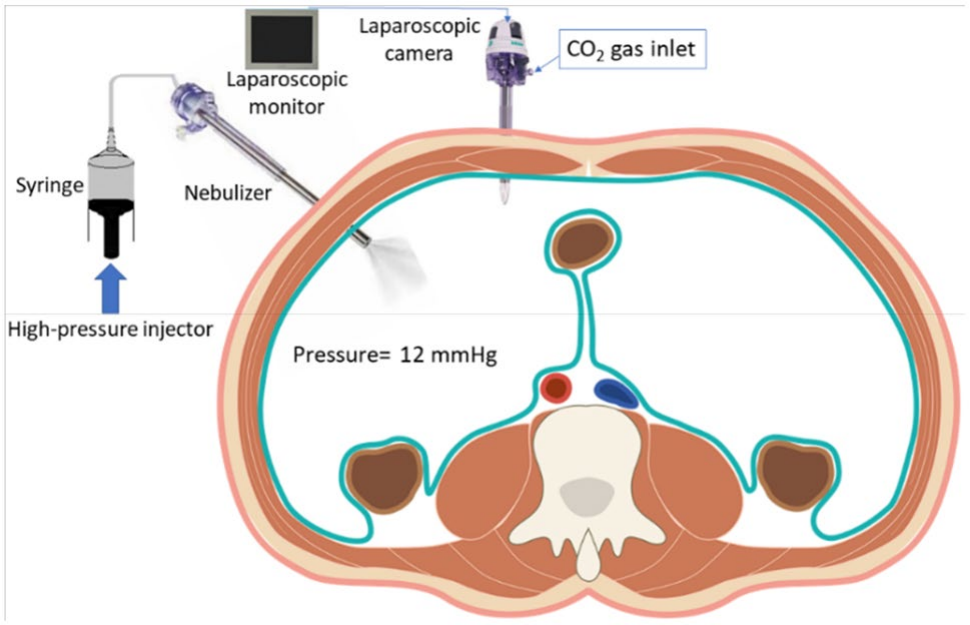
Schematic illustration of pressurized intraperitoneal aerosol chemotherapy (PIPAC).

Pressurized IntraPeritoneal Aerosol Chemotherapy (PIPAC) was recently introduced as an innovative drug delivery method to treat PM^8,9^. PIPAC combines IPDD with a minimally invasive approach (laparoscopy) and it is repeatable. Furthermore, PIPAC may improve drug penetration in tumoral tissue due to an increased intraperitoneal pressure^10^. During PIPAC, a CO_2_ pneumoperitoneum is first established (12 mmHg) to inflate the peritoneal cavity. Then, a drug-containing solution is typically aerosolized within the inflated peritoneal cavity by means of an atomizer (Capnopen®, Capnomed, Zimmern, Germany) and a high-pressure injector (Injektron™ 82 M, Medtron, Saarbrücken, Germany) with the aim to obtain a homogenous aerosol distribution within the peritoneal cavity^11^ (Fig. 1).

Several preclinical (in vitro^9^, ex vivo^12^, and in vivo^8^) and clinical^13,14^ studies demonstrated the feasibility, safety, and efficacy of PIPAC. However, studies have shown that the spatial distribution of the generated aerosol is not uniform: due to gravity and inertial impaction, most of the drug is delivered in the anatomical region which is located opposite from the nozzle of the aerosolizer^15^.

In order to further improve the efficacy of PIPAC, the effects of several treatment parameters such as flow rate, droplet size, addition of an electrostatic field, or treatment duration need to be studied in detail.

Advances in computational speed have lead to an increased adoption of computational fluid dynamics (CFD) models to study aerosol dynamics in a variety of industrial and scientific applications. In the field of respiratory medicine, the use of CFD models in combination with geometrical models of the respiratory tract has been extensively studied as a tool to predict and improve the delivery of therapeutic (inhalation) aerosols^16^. However, to the best of our knowledge, no studies are available that have used CFD models combined with in vitro validation related to *intraperitoneal* aerosol delivery during laparoscopy. There are essential differences between inhalation and IPDD as an aerosol in terms of fluid flow (airflow in airways versus stationary CO_2_ pneumoperitoneum in PIPAC), the relevant anatomy and geometry (fractal airway geometry versus homogeneous peritoneal cavity), and aerosol type (solid particle or liquid droplet).

Here, we used computational fluid dynamics (CFD) modeling in combination with in vitro validation to define the optimal treatment parameters and thus guide clinical PIPAC therapy.

## Materials and methods

### In-vitro experiments

**Figure 2:**
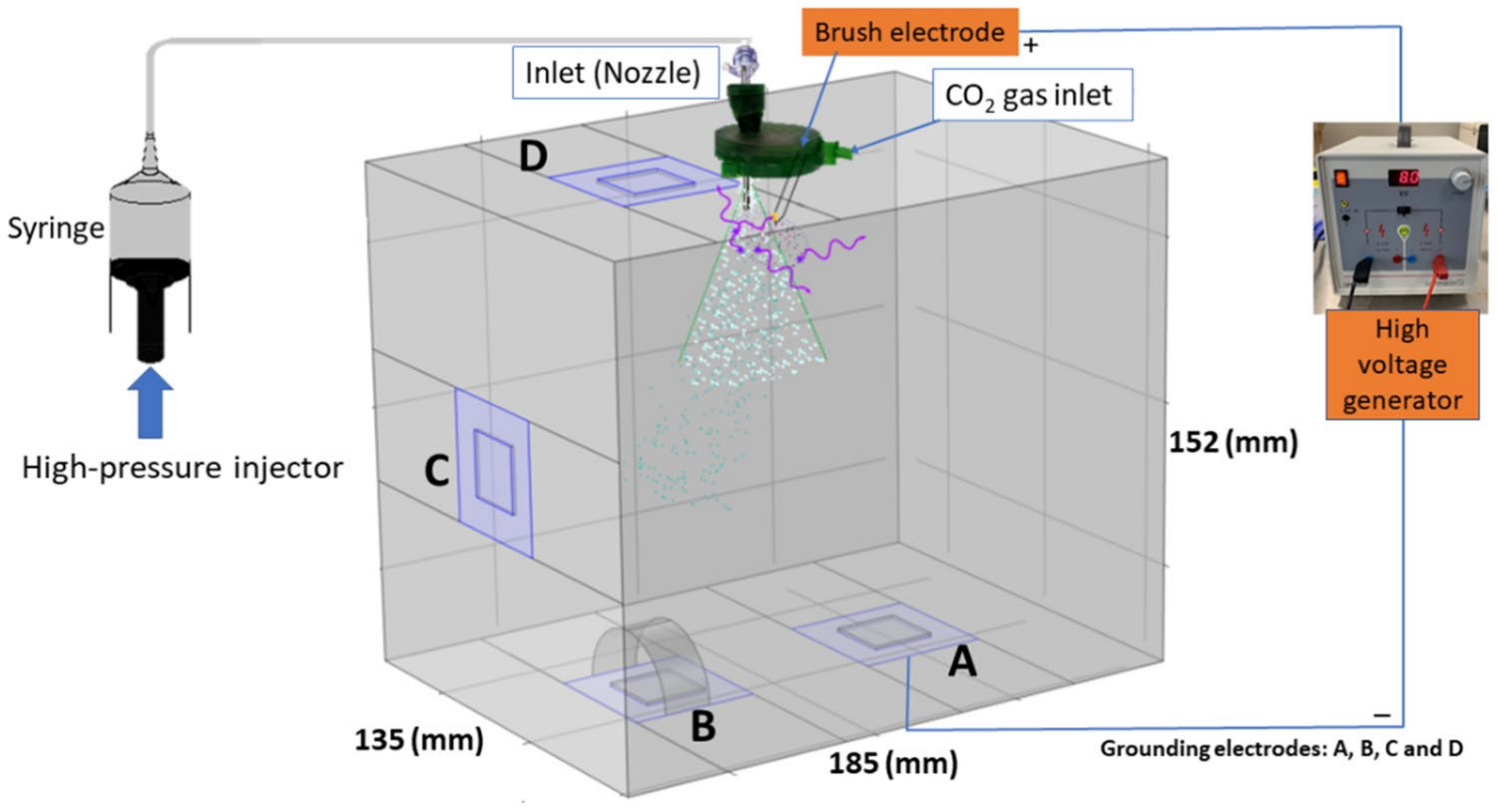
Illustration of the in-vitro ePIPAC box model. A, B, C and D are the positions of the metal plates to hold the tissue samples and grounding plates.

In-vitro experiments were performed using a plexiglass box (185 × 135 × 152 mm^3^, Fig. 2), as previously described^17^. The nebulizer was placed at the top surface of the box through a GelPOINT Mini access platform (Applied Medical, Amersfoort, The Netherlands). A volume of 20 mL of black ink (Pelikan nv, Groot-Bijgaarden, Belgium) was nebulized with a volumetric flow rate (*Q*) of 0.5 mL s^−1^ and upstream pressure of 20 bar using a high pressure injector (Injektron 82 M, Medtron AG, Saarbrücken, Germany). Fresh rat peritoneal tissue was obtained from animals that were culled after neurosurgical experimentation at Ghent University. Tissue samples (approximately 2 mm thick and 2 by 2 cm) were positioned on metal plates located at four different sites in the box to allow visualizing and quantifying the distribution of black ink aerosol droplets: on the bottom surface without any additional obstacle (A), under a curved plastic obstacle on the bottom surface (B), on the side surface (left) (C), and on the top surface (D). The samples were positioned with the peritoneal surface facing the aerosol, and the skin surface in contact with the metal plates. A pressure regulated insufflator was used to create a CO_2_ pressure of 12 mmHg (1600 Pa) in the box prior to black ink nebulization. After each experiment (with a duration of 30 min), digital images were obtained to document ink coverage of the tissue samples. Subsequently, the extent of ink coverage and distribution were calculated by means of threshold functions (brightness 1–100) with ImageJ software (version 1.51, National Institutes of Health, Bethesda, Maryland, United States, available from https://imagej.nih.gov/ij/index.html). A region of interest (ROI) was drawn around the tissue border and the area of the ROI was measured. The proportion (%) of stained tissue surfaces was calculated as follows:

$$Proportion\,stained\,ROI \left(\mathrm{\%}\right)=\frac{Area\,of\,black\, stained\,surface\,in\,ROI}{Total\,area\,in\,ROI}\times 100$$

The measurements were performed in triplicate.

### Characteristics of aerosol droplets

#### Aerosol droplet size measurement

Measurements of the volume weighed particle size distribution (PSD) of aerosol droplets was performed using laser diffraction (Mastersizer S long bench, Malvern Instruments, Malvern, United Kingdom). An open laser beam (water vs. air, refractive index of 1.33 and 1.00, respectively) was created with a 300F lens (0.5–900 μm) after 10 s of nebulization. The laser diffraction instrument obtains the size distribution of aerosol droplets by measuring the angular variation in intensity of light scattered as a laser beam passes through the droplets. When the aerosol droplets interfere with the laser beam, they create a diffraction pattern. Droplet size measurements were carried out at 3.5 and 10 cm from the tip of the nozzle and the lens, respectively. Several experiments were performed to cover a range of flow rates (0.4, 0.5, 0.6, 0.7, 0.8 and 0.9 mL s^−1^). Results were exported as the median of the volume distribution, D(v,0.5), i.e. the volume median diameter at which 50 vol% of the aerosol droplets were either finer or coarser than the predicted value, with standard deviation.

#### Cone angle of nebulization

The nozzle geometry and characteristics affect the aerosol droplet behavior during nebulization^18^. The diameter of the orifice and the cone angle of nebulization are two important characteristics of the nozzle. The cone angle has a relationship with the driving pressure, flow rate, and viscosity of the fluid. In the current PIPAC technique, the high-pressure nozzle has an orifice with a diameter of 200 µm. The experiments were performed using water and flow rates of 0.2, 0.5, 0.8 and 1.1 mL s^−1^ with a maximal pressure of 20 bar. During each experiment, digital images were obtained during nebulization and imported to ImageJ to measure the cone angle. During CFD simulation, the cone angle was determined directly by COMSOL Multiphysics (Measure accumulator) by defining two edges drawn in a plane from the nozzle tip to the outer periphery of the aerosol.

#### Viscosity measurement

In order to investigate the effect of the viscosity of the carrier liquid, we measured the viscosity of Icodextrin (Baxter Healthcare Ltd, Illinois, US), a glucose polymer preparation, at concentrations of 4% and 7.5% using a capillary viscometer (Paragon Scientific Ltd, Birkenhead, UK). The procedure was performed according to the guidance from the European Pharmacopoeia 10.0^19^. Saline solution (NaCl 0.9%) was used to calibrate of the viscometer. The experiments were performed in triplicate.

#### Electrostatic precipitation

To model electrostatic precipitation, we used a high voltage power supply (LD Didactic, Hürth, Germany, 0–10 kV, current 2 mA). An active cable with a steel brush electrode and four return electrodes connected to four metal grounding plates were added to the box model (Fig. 2). The electrostatic precipitation was initiated from the start of nebulization.

### Computational approach

#### Geometry and mesh generation of the box model

**Figure 3:**
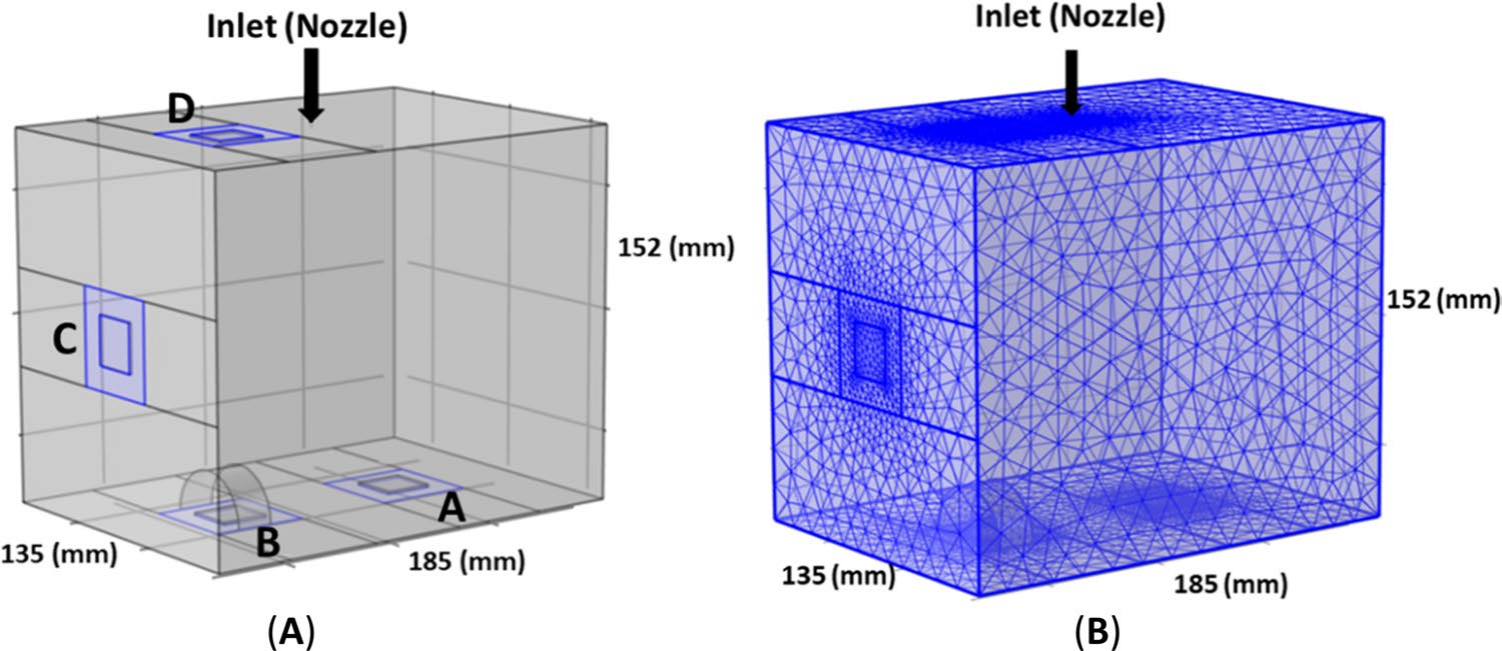
Illustration of the preparation of the simulation geometry for the CFD simulation approach: (**A**) 3D box model geometry, and (**B**) mesh generation.

A 3D model of a rectangular box (185 × 135 × 152 mm^3^) (Fig. 3A) was created using COMSOL Multiphysics (COMSOL, Inc., Burlington, VT, USA). The fluid domain within the box geometry was spatially discretized using tri/tetrahedral grids with local refinements near the nozzle inlet and locations A-D (Fig. 3B). To ensure valid results, a mesh sensitivity study was performed, resulting in an optimal volume mesh containing 98,798 tetrahedral elements.

**Figure 4:**
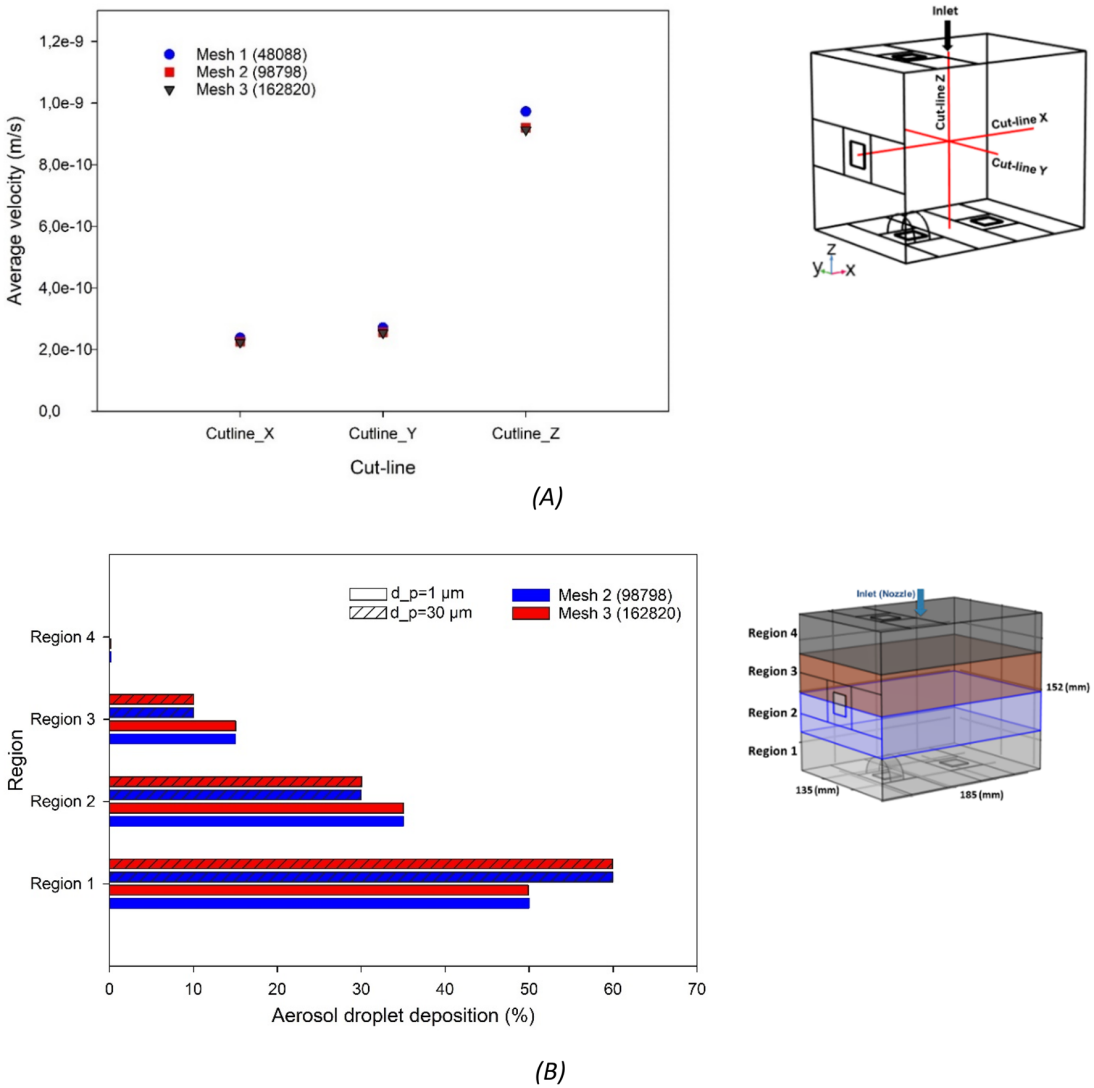
Mesh independence test based on, (**A**) the average velocity of the CO_2_ phase after reaching 12 mmHg along three cut-lines (X, Y and Z directions), and (**B**) deposition of aerosol droplets in four regions of the in vitro box model for two different droplet diameters (1 and 30 µm;

The physical domain was discretized into a large number of tri/tetrahedral computational elements with local refinements near critical locations, i.e. near the nozzle inlet and near the tissue holding plates inside the box. The mesh quality was evaluated by examining the skewness measure of mesh elements which is based on equiangular skew. It was assumed that a skewness is less than 0.9 was acceptable. The maximum skewness ranged from 0.68 to 0.85, in range with published recommendations^20^. Elements with a quality below 0.1 were considered as a poor quality mesh. To ensure valid results, a mesh sensitivity study was performed, resulting in an optimal hybrid volume mesh containing 98,798 tri/tetrahedral elements. The model was tested for three different grid densities, i.e. containing 48,088 (Mesh 1), 98,798 (Mesh 2) and 162,820 (Mesh 3) volume elements, by comparing the CO_2_ phase stability after reaching a pressure of 12 mm Hg. Three cut-lines were considered in the X, Y and Z directions inside the box model. The average velocity of the CO_2_ phase in these three directions inside the cavity was selected as one of the criteria for mesh independency. The comparison of the average velocity of CO2 phase for all three grid resolutions is provided in Fig. 4A. Comparing the results showed a 5.6% difference between Mesh 1 and Mesh 2, and a 0.7% difference between Mesh 2 and Mesh 3. The increase of element number from 98,798 to 162,820 grids did not change the results. Additionally, to investigate the effects of mesh resolution on aerosol droplet distribution, the droplet deposition efficiency of 2 mesh densities, 98,798 (Mesh 2) and 162,820 (Mesh 3) elements, were compared for 4 regions of the in vitro box model (regions 1–4) for two different droplet diameters (1 and 30 µm). Comparison of the droplet deposition percentage over specific regions (region 1, 2, 3, and 4) of two mesh densities showed that the maximum error was 0.3% (Fig. 4B). Therefore, we adopted the mesh that contained 98,798 grid cells, the combination that gave the best grid independency and stability in trade-off with the computational cost. The CFD simulations were performed on the high performance computing infrastructure of Ghent University, and on a Dell Precision 5820 workstation with 256 GB RAM and 4.00 GHZ CPU, resulting in typical calculation times of 48–120 h.

#### Continuous phase modeling (CO_2_ gas)

As a first step, the CFD approach filled the virtual box with CO_2_ gas to a pressure of 12 mmHg (1600 Pa). The governing equations of the CO_2_ phase were considered under unsteady, laminar, incompressible flow and Newtonian fluid assumptions. The associated continuity and momentum (Navier–Stokes) equations can be expressed as follows using Einstein notation^21^:

- Continuity equation
  1 $$\frac{\partial {u}_{i}}{\partial {x}_{i}}=0$$
  where *u*_*i*_ [m s^−1^] (i = 1, 2, 3) are the flow velocity components, and *x*_*i*_ [m] denotes the droplet position components.
- Momentum equation
  2 $$\frac{\partial {u}_{i}}{\partial t}+{u}_{j}\frac{\partial {u}_{i}}{\partial {x}_{j}}=-\frac{1}{{\rho }_{0}}\frac{\partial p}{\partial {x}_{i}}+\frac{\partial {\tau }_{ij}}{\partial {x}_{j}}$$
  where ${\tau }_{ij}=\nu (\frac{\partial {u}_{i}}{\partial {x}_{j}}+\frac{\partial {u}_{j}}{\partial {x}_{i}})$ [Pa] is the viscous stress related to incompressible flow, *ν* [m^2^ s^−1^] the fluid kinematic viscosity, *p* [Pa] the static pressure, and *ρ*_*0*_ [kg m^−3^] the fluid density. The inflow boundary condition for CO_2_ gas was assumed to be a pressure inlet (12 mmHg = 1600 Pa) at the level of the nozzle indicated on Fig. 3. A no slip condition was used at the rigid walls. The Laminar Fluid Flow physics submodule within the CFD module in COMSOL Multiphysics™ was used to simulate the CO_2_ inflow (*ρ* = 1.977 kg m^−3^; *ν* = 7.44*10^–6^ m^2^ s^−1^).

#### Discrete phase modeling (aerosol droplet)

After a stable pneumoperitoneum of 12 mmHg was created by means of the CO_2_ phase modelling, the second step of the CFD approach focused on analyzing aerosol droplet transport using the Particle Tracing for Fluid Flow module in COMSOL Multiphysics. Nebulization of a volume of 20 mL of black ink (density: 1070 kg m^−3^, dynamic viscosity: 2.1 mPa s) was simulated with a volumetric flow rate of 0.5 mL s^−1^ at a fixed injector position defined as an inlet at the middle of the top surface of the box. Aerosol droplets were injected during 40 s, and the total simulation time was 30 min. Based on the flow rate (*Q* = 0.5 mL s^−1^) and the area of the orifice (*A*_*O*_) of the nebulizer nozzle (diameter = 200 µm), the initial velocity (*u*_*0*_) was calculated as 15.92 m s^−1^ (*u*_*0*_ = *Q/A*_*O*_). The droplet diameter was set at 30 µm^22^. Impacted droplets were assumed to adhere to the sidewalls if the distance between the droplet center and the sidewall was less than the droplet’s diameter.

#### Two-phase model

The simulation entailed a discrete (aerosol droplets) and continuous (gas) phase. Aerosol transport was modelled by tracking individual droplets as they move through the CO_2_ gas using a Lagrangian approach^23^. Based on the volume fraction of the discrete phase, the two-phase flow can be described using either one-way or two-way coupling. The volume fraction is given by^24,25^:

3 $$\varphi =\frac{\sum_{i=1}^{N}{v}_{i}}{V}$$

where *N*, *v* and *V* are the number of droplets, droplet volume, and the volume of gas (continuous phase) respectively. In the present study, the volume fraction ($\varphi$) is less than 10^–6^, indicating the continuous phase (CO_2_ gas) significantly outweighs the discrete phase (aerosol droplets). Using this approach, the two-phase flow can be considered as dilute and the momentum imparted onto the fluid by the droplets can be neglected, justifying the assumption to use one-way coupled simulations^26,27^.

The simulation was solved in two stages. First, the CO_2_ gas flow (continuous phase) was solved using a stationary study step. Then, the aerosol droplet (discrete phase) trajectories were computed using a time dependent study step. The solution from the stationary study was used to define the CO_2_ pressure and velocity for the purpose of exerting a drag force on the droplets. The presence of droplets was not considered when modelling the CO_2_ filling of the cavity.

#### Forces acting on individual aerosol droplets

Newton’s second law describes the aerosol droplet transport in the Lagrangian formulation using a Cartesian coordinate system (for a given direction) as follows^28^:

4 $${m}_{p}\frac{\partial {{\varvec{u}}}_{p}}{\partial t}={{\varvec{F}}}_{D}+{{\varvec{F}}}_{G}+{{\varvec{F}}}_{E}$$

where *m*_*p*_ [kg] and *u*_*p*_ [m s^−1^] are the droplet mass and velocity, while ***F***_*D*_ [N], ***F***_*G*_ [N] and ***F***_*E*_ [N] are the drag, gravitational and electrical forces, respectively. ***F***_*D*_ is a resistance force related to the aerosol droplet characteristics (size and density) and the fluid viscosity. The resulting ***F***_*D*_ on a droplet with a diameter *d*_*p*_ is defined by Stokes’s equation as follows:

5 $$\left\{\begin{array}{ll}{{\varvec{F}}}_{D}=\frac{1}{{\tau }_{p}}{m}_{p}\left({\varvec{u}}-{{\varvec{u}}}_{p}\right)\\ {\tau }_{p}=\frac{{\rho }_{p}{d}_{p}^{2}}{18\mu }\end{array}\right.\to {{\varvec{F}}}_{D}=3\pi \mu {d}_{p}\left({\varvec{u}}-{{\varvec{u}}}_{p}\right)$$

where *µ* [mPa s] and *u* [m s^−1^] are the viscosity and velocity of CO_2_ gas, and *ρ*_*p*_ [kg m^−3^] is the density of an aerosol droplet. *τ*_*p*_ [s] is the aerosol droplet relaxation time scale. In general, *τ*_*p*_ is dependent on the droplet’s Reynolds number *Re*_*p*_ defined as

6 $${Re}_{p}=\frac{{\rho }_{p}\left|{\varvec{u}}-{{\varvec{u}}}_{p}\right|}{\mu }$$

since the *Re*_*p*_ number affects the inertial impaction and drag force.

The gravitational force (***F***_*G*_) due to gravitational acceleration (***g*** [m^2^ s^−1^]) can be expressed as follows:

7 $${{\varvec{F}}}_{G}={m}_{p}\mathbf{g}=\frac{1}{6}{\rho }_{p}\pi {d}_{p}^{3}\mathbf{g}$$

When adding electrostatic precipitation, an additional electrical force (***F***_*E*_) is generated by the electrostatic field, which is proportional to the electrical charge of the aerosol droplets (*q* [C]) and the strength of the electrical field (***E*** [V m^−1^]):

8 $${{\varvec{F}}}_{E}=q {\varvec{E}}$$

The overall force balance (Eq. 4) can be rewritten in Cartesian coordinates as follows:

9 $${m}_{p}\frac{\partial {{\varvec{u}}}_{p}}{\partial t}={m}_{p}\frac{{\varvec{u}}-{{\varvec{u}}}_{p}}{{\tau }_{p}}+{m}_{p}\mathbf{g}+q {\varvec{E}}$$

#### Stokes and Weber numbers

When a volume of fluid is forced through a nozzle orifice, the pressure head of the fluid is converted into kinetic energy. The probability of deposition by inertial impaction is higher when the droplets are more likely to travel longer distances, which is based on the velocity and the size of the droplet. The Stokes number (*Stk*) is the ratio of the aerosol droplet stopping distance (${\tau }_{p}=\left|{\varvec{u}}-{{\varvec{u}}}_{p}\right|$) and half of the characteristic length (0.5 *d*_*N*_) as follows^11,29^:

10 $$\genfrac{}{}{0pt}{}{Stk=\frac{{\tau }_{p}}{{t}_{f}}=\frac{{\tau }_{p}\left|{\varvec{u}}-{{\varvec{u}}}_{0p}\right|}{{0.5 d}_{N}}}{\left({Re}_{p}\ll 1\right) , {t}_{f}=\frac{{0.5 d}_{N}}{\left|{\varvec{u}}-{{\varvec{u}}}_{0p}\right|}}$$

where *t*_*f*_, *d*_*N*_ and *u*_*0p*_ are the characteristic flow time scale [s], nozzle diameter [m] and aerosol droplet initial velocity [m s^−1^], respectively.

The Weber number (*We*) is the ratio between the inertial and the surface tension forces of liquid^30^:

11 $$We=\frac{{\rho }_{p}{\left|{\varvec{u}}-{{\varvec{u}}}_{p}\right|}^{2}{d}_{p}}{\sigma }$$

where σ [N m^−1^] is the surface tension. The *We* number indicates whether the kinetic or the surface tension energy is dominant: the higher the *We* number, the more dominant is the kinetic energy^30^. The value of the *We* number determines the mode of droplet breakup, reflecting the minimal initial inertial force required to cause droplet breakup assuming a certain restoring force, under an impulsive acceleration. The *We* number characterizes the tendency of the liquid to break up under competing inertia and surface tension forces.

#### In silico simulation

For CFD simulation of aerosol droplet transport, the same steps were applied in silico: insufflation of CO_2_ to reach a pressure of 12 mmHg, followed by nebulization of ink. The computational domain was assumed to be incompressible. For the in vitro validation experiment and simulation of the box model, the internal pressure was increased by injecting CO_2_ to resemble the time point at which the cavity is fully inflated at a steady state pressure of 12 mmHg. In this situation, the peritoneal cavity can be assumed to be an incompressible domain. Then, a drug-containing solution was aerosolized within the (inflated) peritoneal cavity by means of an atomizer.

The CFD and Particle Tracing Modules were applied under appropriate initial and boundary conditions. The dimensions of the simulated tissue samples at locations A-D (see Fig. 3A) were 20 × 20 × 2 mm^3^. The locations of tissues in the in-vitro experiment and in-silico identical (A, B, C and D). Tissue properties (conductivity, density, Young’s modulus, relative permeability and electrical conductivity) were defined using the material library model for human tissue in COMSOL Multiphysics. In order to estimate the deposition of aerosol on the simulated tissue samples, the following procedure was used to mimic the experimental (in vitro) setup: after running the simulation, the areas representing the virtual samples were selected and exported as a TIFF file. Using these files, a threshold function was applied to select the stained pixels, and the stained proportion was calculated using Image J. The number of deposited aerosol droplets in different regions of the box model was measured with a Particle Counter Accumulator. In the simulation, unsteady particle tracking was used with injection time step size of 0.001 s for a total duration of 40 s to inject the droplets as a random distribution.

### Parameter study

Given the lack of computational studies of the PIPAC technique, we investigated the influence of different parameters on aerosol droplet behavior. A baseline CFD model was generated using typical PIPAC parameter values (*d*_*p*_ = 30 µm and *Q* = 0.5 mL s^−1^)^22^. Subsequently, a parameter study was performed to vary the aerosol droplet diameter (*d*_*p*_ [µm]), flow rate of nebulization (*Q* [mL s^−1^]), and liquid viscosity (*µ* [mPa s]). The simulations allowed to determine the effect of these parameters on the deposition of the aerosol droplets in different regions of the model. Condensation and evaporation effects were neglected for the PIPAC simulations. In pulmonary medicine, the elevated temperature and relative humidity of the airways are known to potentially affect the properties of aerosol droplets by causing evaporation, condensation, and hygroscopic growth^31^. However, using current laparoscopic technology, cold and dry CO_2_ gas, which has a very low relative humidity (typically 0.0002%), is insufflated at room temperature (20 °C). Therefore, these two phenomenona were assumed to be negligible during PIPAC.

The simulation was solved in two stages. First, the CO_2_ gas flow (continuous phase) was solved for using a stationary study step. Then, the aerosol droplet (discrete phase) trajectories were computed using a time dependent study step. The solution from the stationary study was used to define the CO_2_ pressure and velocity for the purpose of exerting a drag force on the droplets. The presence of droplets was not considered when modelling the CO_2_ filling the cavity. A second-order (quadratic) discretization of fluid flow was used and the partial derivative equations were solved with a dominant second derivative term. A fully coupled method was applied to generate the single set of algebraic equations for all the involved physical models, and implemented in a single iteration scheme which was repeated until convergence was reached. The model was considered converged when the estimated error in the iterative solver was below 10^–6^.

## Results and discussion

### Experimental validation of the CFD model

**Figure 5:**
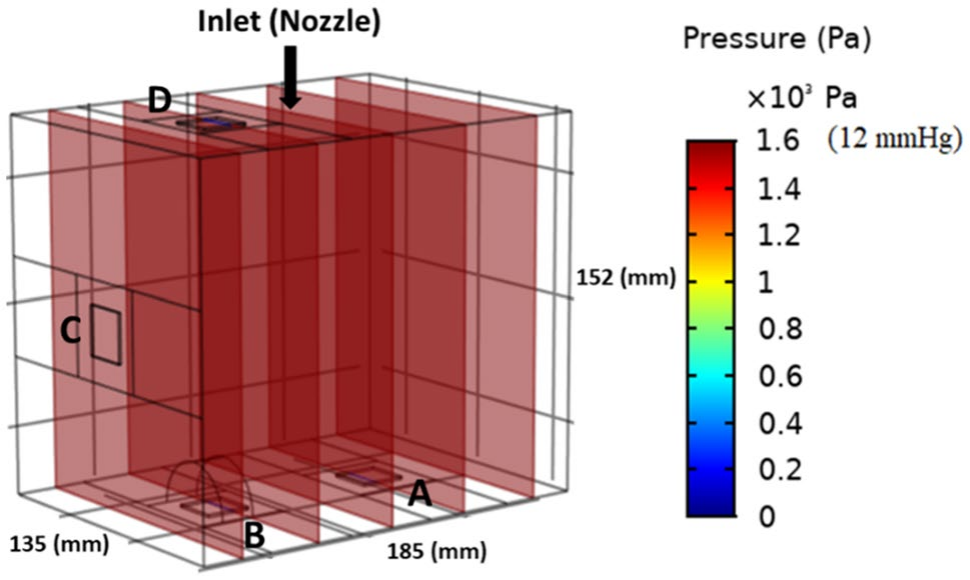
CFD simulation result of CO_2_ gas filling of the box model to a pressure of 12 mmHg.

**Figure 6:**
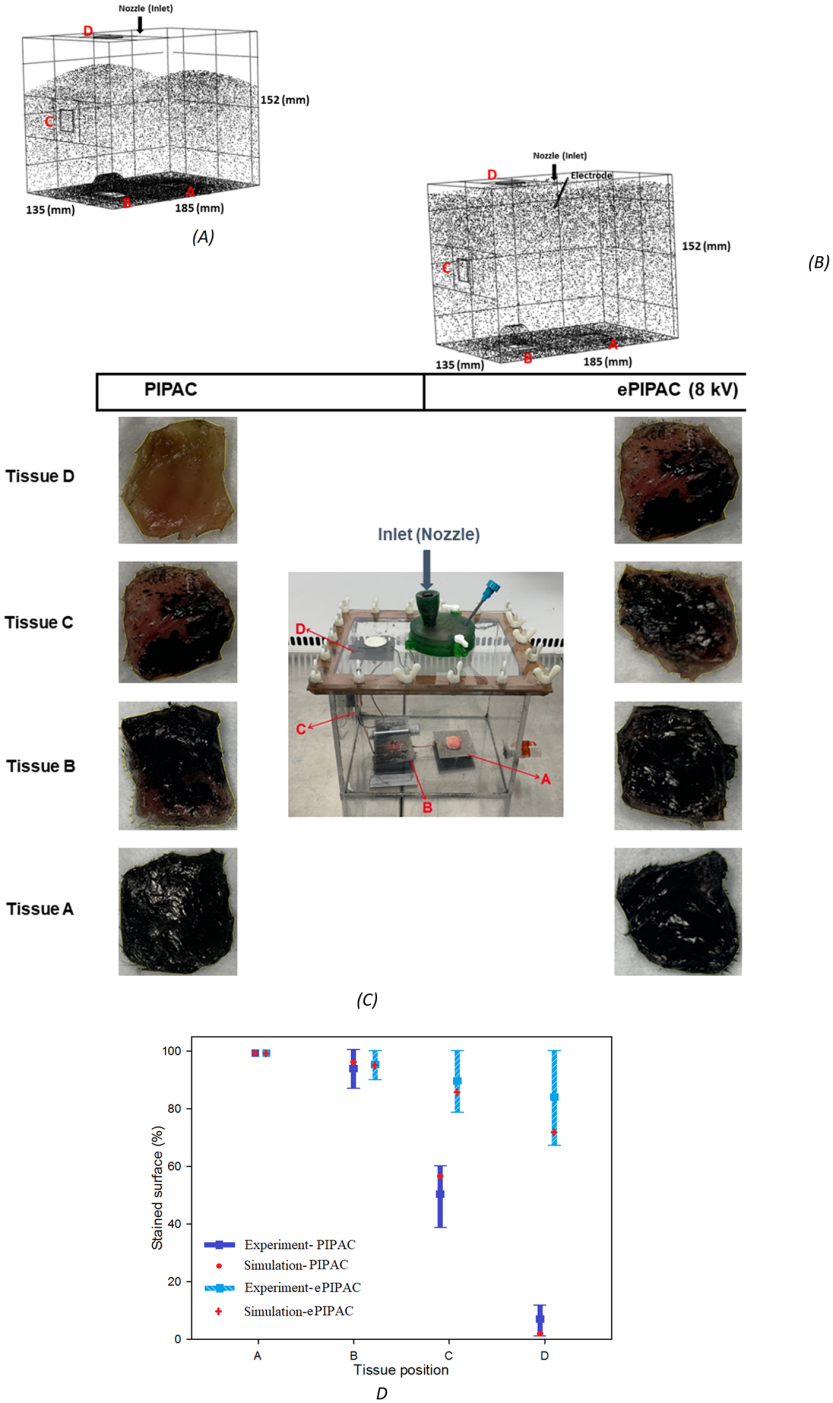
Aerosol droplet distribution patterns in the box model obtained using CFD simulations for (**A**) PIPAC and (**B**) ePIPAC. (**C**) typical aerosolized ink deposition patterns on the tissue samples located at different locations in the in vitro box model, with and without application of electrostatic precipitation. (**D**), comparison between in vitro results and CFD simulation of aerosol distribution at positions A–D. In vitro experiments were performed in triplicate; error bars represent mean (SD).

In a first step, we modelled filling of the box with CO_2_ gas to a pressure of 12 mmHg (1600 Pa), Fig. 5. Next, we simulated the spatial distribution of aerosol droplets after nebulization for PIPAC and ePIPAC (Fig. 6A,B). As expected, the simulation showed that most aerosol droplets were deposited at the bottom region of the box due to gravity and inertial impaction during PIPAC. Figure 6C,D display the droplet distribution after experiments of PIPAC and ePIPAC, respectively. As can be seen, the spatial distribution of droplets after ePIPAC is more homogenous than PIPAC in the box model Fig. 6E compares the percentage of tissue surface ink staining (regions A-D) obtained in vitro with the CFD simulation, showing an overall strong agreement between both. Tissue at the bottom surface was almost completely covered by black ink after PIPAC. Tissue on plate C was partially stained, while staining of tissue on plate D was very limited. By imposing an electrostatic field in the box to mimick ePIPAC, the aerosol distribution improved and was more homogenous. Both the experiment and simulation results show a significantly better aerosol droplet deposition at the top of the box model (plate D) when an electrical force is created.

### Effect of aerosol droplet size

The aerosol droplet size is a main parameter affecting the type of forces that impact aerosol droplet transport^32^. As mentioned in “Stokes and Weber numbers” (Eq. 10), the *Stk* number, is a measure of the influence of the inertial effects during droplet transport. Thus, *Stk* as well as *Re*_*p*_ have to be considered carefully to capture more accurately the underlying physics of droplet deposition. Understanding the relationship between the aerosol droplet size and forces is helpful to predict the behavior of the aerosol droplet. As shown in Eq. (5), the drag force has a direct relationship with the aerosol droplet diameter. Also, the gravitational force has a direct relationship with the aerosol droplet diameter to the 3rd power (see Eq. 7). When the size of the aerosol droplet raises, its mass and subsequently the gravitational force also significantly increase. Thus, for large aerosol droplets, drag and especially gravitational forces play a significant role^32^.

**Figure 7:**
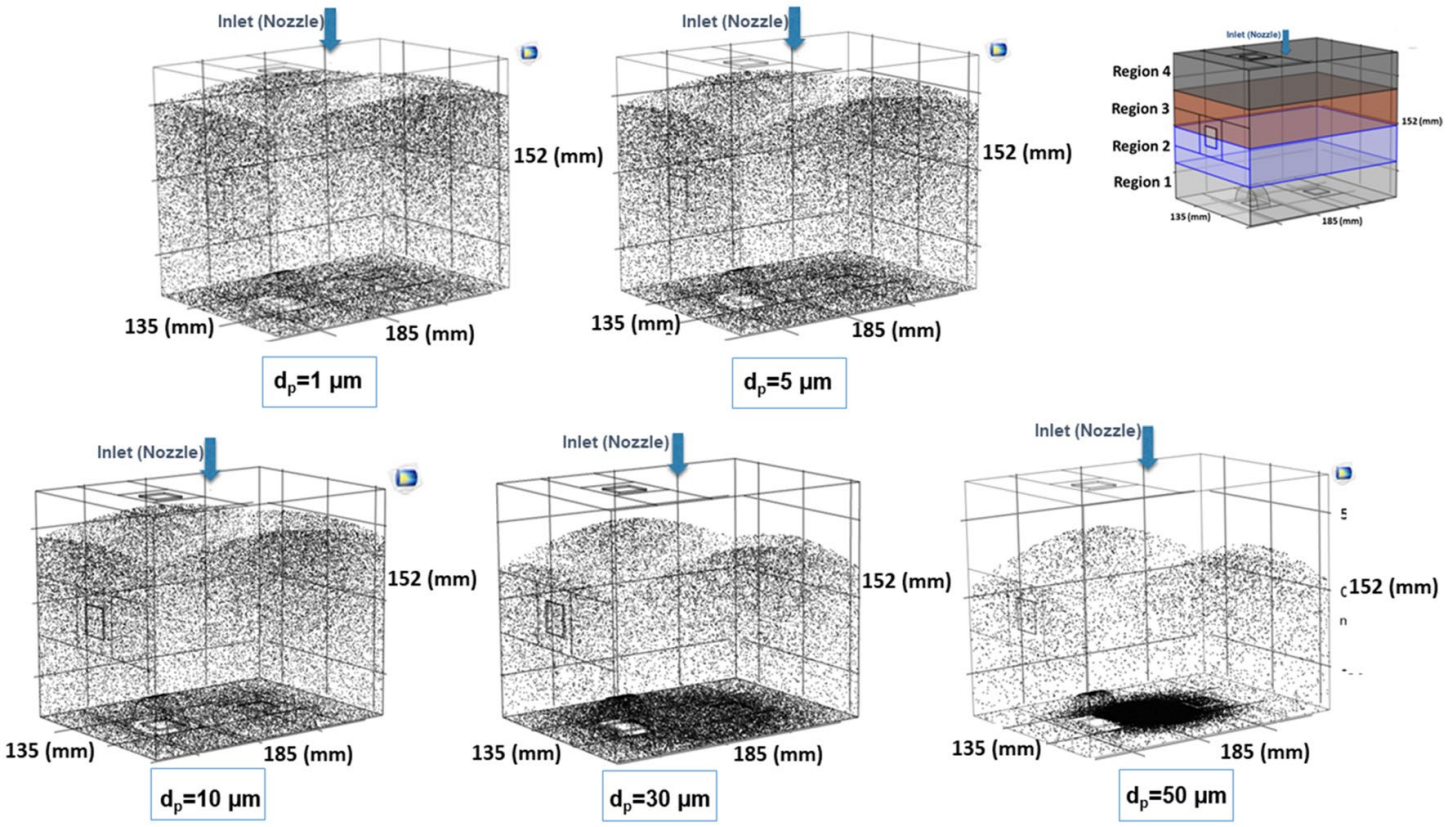
Nebulization of black ink at a flow rate of 0.5 mL s^−1^ for different aerosol droplet diameters using CFD. The black dots display the aerosol droplets.

**Figure 8:**
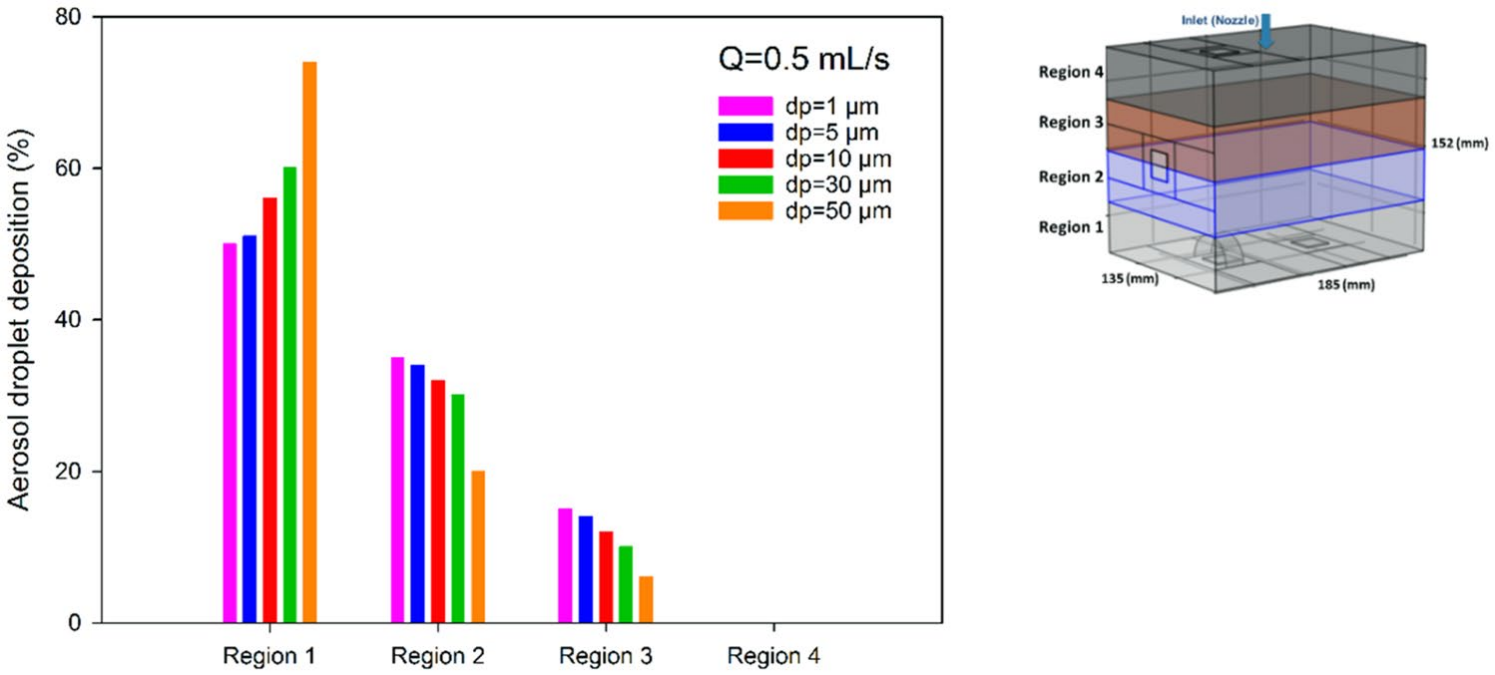
Comparison of the simulated deposition of the aerosol droplets with different diameters (d_p_) in four regions in the CFD box model. Deposition in region 4 (ventral) was negligible.

For the analysis of the effect of aerosol droplet size, the CFD modelled box was divided in four regions, corresponding to dorsal (region 1) to ventral (region 4) regions of the peritoneal cavity. Five different aerosol droplet diameters were modelled (see Fig. 7). The simulations showed that aerosol droplet deposition is significantly affected by their diameter. As shown in Fig. 7, the effect of gravity is quite significant for droplets in the range of 30–50 µm. For smaller droplets, gravity has only a small effect on the deposition pattern. Figure 8 shows the simulated deposition of aerosol droplets, according to diameter, in the four defined regions. With increasing diameter, most droplets deposited in the dorsal region (region 1). With a diameter of 30 µm, 60% of the aerosol droplets deposited in region 1, 30% in region 2, 10% in region 3, and none in region 4. With a diameter of 50 µm, 78% of aerosol droplets deposited in region 1. The results showed that a droplet diameter ranging between 1 and 5 µm resulted in the most homogenous spatial distribution, although coverage of the most ventral region (region 4) remained virtually absent with this flow rate, due to less perturbation of aerosols. More perturbation in the aerosol droplet flow results a better distribution.

### Effect of liquid flow rate

#### Fixed droplet diameter

**Figure 9:**
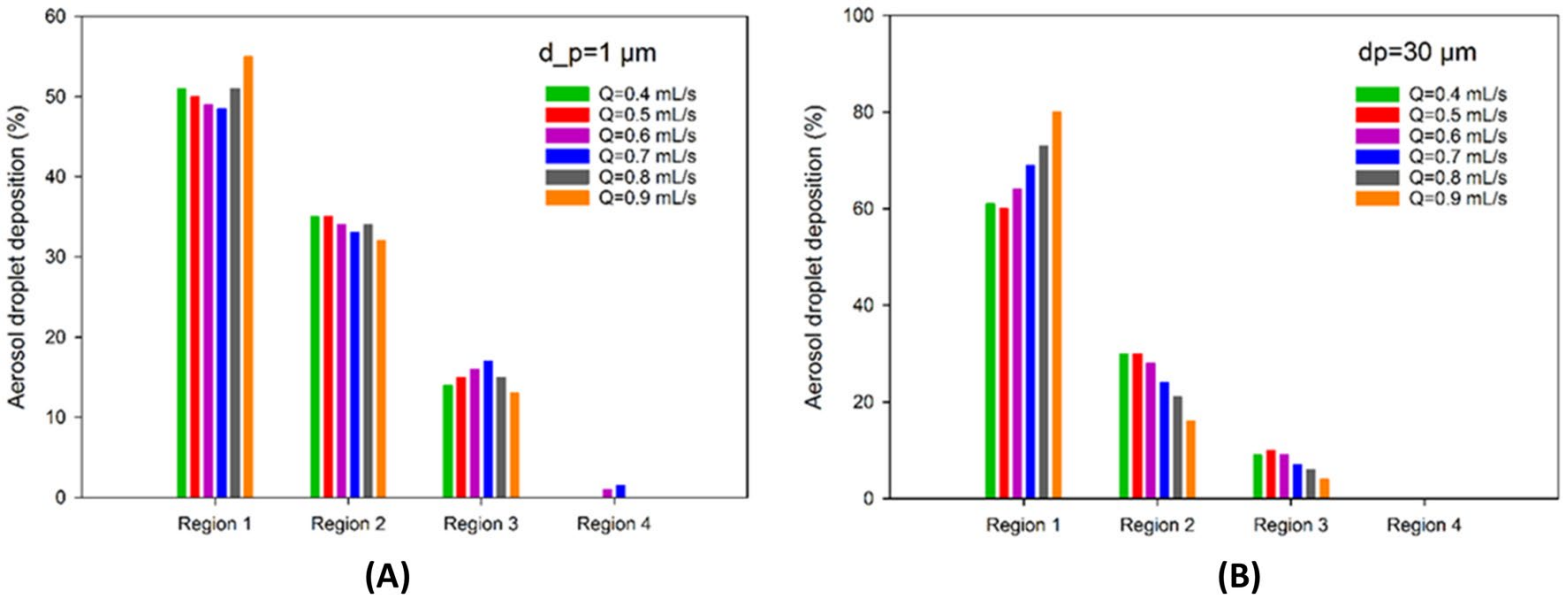
Comparison of aerosol deposition with varying liquid flow rate (Q) in four regions of the CFD box model, assuming a droplet diameter of (**A**) 1 µm, and (**B**) 30 µm.

Figure 9A,B compare the deposition of the aerosol droplets with different flow rates in four regions of the box using CFD simulations. In these comparisons, the simulation was done for two different diameters, while all other properties except flow rate of the nebulization were kept constant. For a constant droplet size, increases in flow rate led to a higher initial velocity, concomitant increases in inertial impaction and kinetic energy, and extensive deposition in region 1 (opposite the nebulizer). However, very low flow rates (< 0.4 mL s^−1^) also resulted in deposition of a large majority of the droplets in region 1. This might probably explained by the inability of the low flow rate and energy to break up the liquid into droplets, and by the dominant effect of gravity, especially for heavy and large droplets, leading to sedimentation^33^. Also, low flow rates tend to decrease the nebulization angle (see “Effect of liquid flow rate on nebulization cone angle”). Inertial impaction and gravitational force seem to have the most effect on the droplet behavior. For a smaller diameter (1 µm), homogeneity of aerosol deposition was found to be optimal with a flow rate of 0.7 mL s^−1^. For diameter of 30 µm, the inertial impaction and gravitational force are higher, and a flow rate of 0.5 mL s^−1^ shows a better distribution of droplets in the box model. Obviously, for larger droplets, the large majority of droplets are deposited on the opposite site of the nebulizer. According to Fig. 9A, due to more complex aerosol flows, some aerosol droplets deposited at region 4 for flow rates of 0.6 mL s^−1^ and 0.7 mL s^−1^. However, for larger droplets, this region remained unexposed.

**Figure 10:**
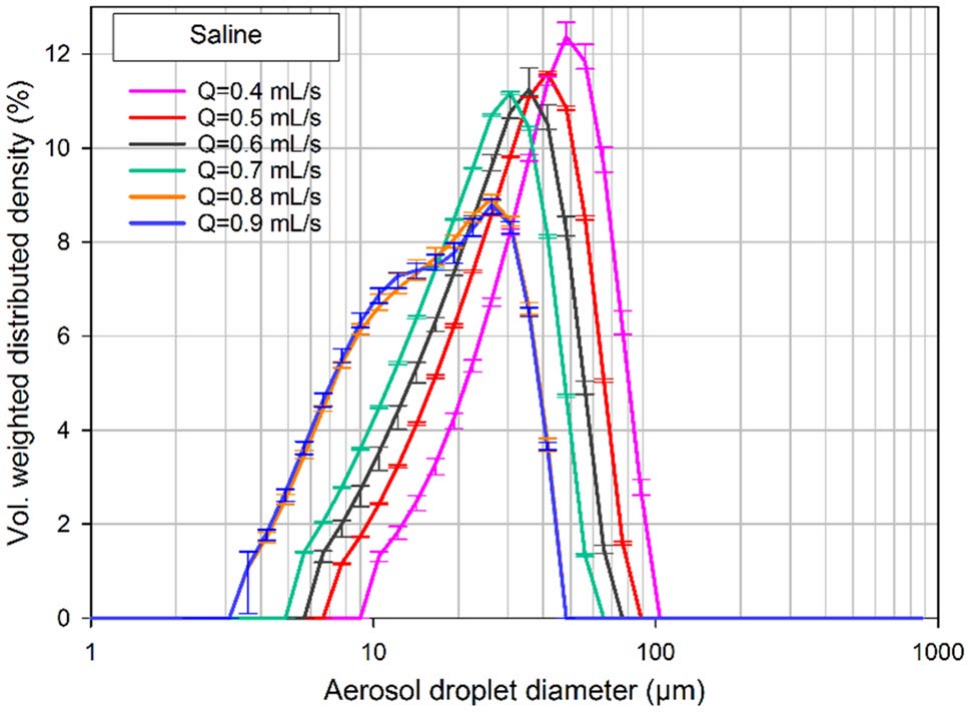
Volume-weighted distributed density of the aerosol droplets showing the effect of flow rate on the droplet size. A range of flow rates between 0.4 and 0.9 mL s^−1^ was used. The nebulization was performed in an open space at an upstream maximum pressure of 20 bar.

Figure 10 demonstrates the experimentally measured volume-weighted distributed density of the aerosol droplet diameters for different flow rates of the nebulization. Interestingly, analysis of different flow rates at a fixed maximal upstream injection pressure of 20 bar, showed that higher flow rates of liquid resulted in a decrease of the volume median diameter of the droplets. When increasing the flow rate, D(v,0.5) values were measured as 48 ± 2 µm (p < 0.0001), 40 ± 1 µm (p < 0.05), 35 ± 2 µm (p < 0.0001), 29 ± 2 µm (p < 0.0001), 28 ± 2 µm (p < 0.05) and 28 ± 3 µm (p < 0.05) for flow rates of 0.4, 0.5, 0.6, 0.7, 0.8 and 0.9 mL s^−1^, respectively.

#### Variable droplet diameter

**Figure 11:**
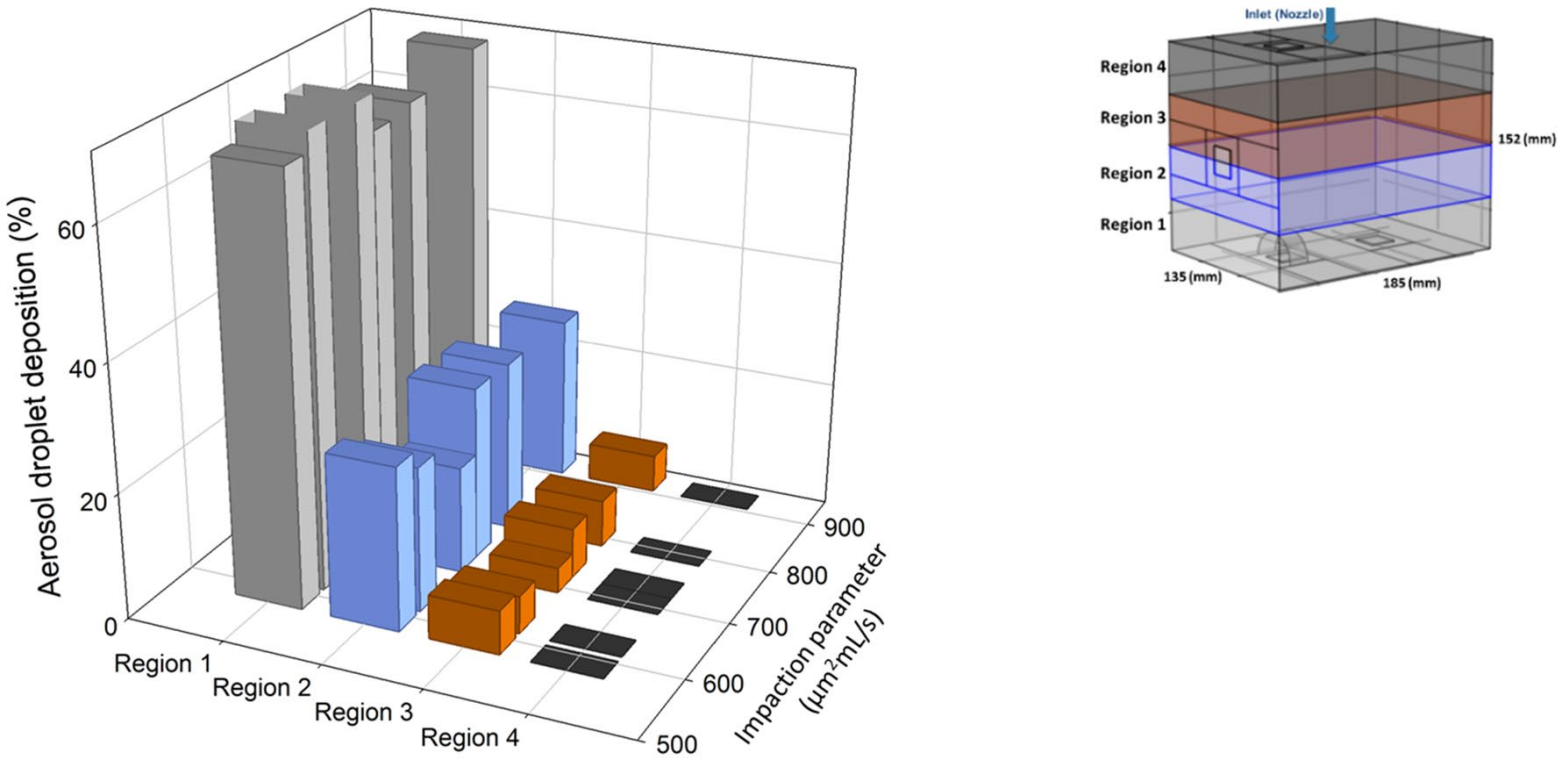
Comparison of the simulated aerosol droplets deposition in four regions of the CFD box model for different values of the impaction parameter. Each bar represents the deposition percentage of the aerosol droplets in the box model.

The magnitude of inertial impaction depends on the droplet diameter and liquid flow rate. As proposed by Cheng et al., inertial impaction can be expressed as an impaction parameter, defined as *d*_*p*_^2^*Q, with *d*_*p*_ = aerosol droplet diameter [µm] and *Q* = liquid flow rate [mL s^−1^]^34^. Table 1 shows impaction parameter values for six different flow rates of nebulization (*Q* = 0.4, 0.5, 0.6, 0.7, 0.8 and 0.9 mL s^−1^). Figure 11 depicts a comparison of the simulated aerosol droplet deposition in the four regions of the CFD box model for different values of the impaction parameter (*d*_*p*_^2^*Q). Each bar represent the deposition percentage of the aerosol droplets in the box model. It shows preferential accumulation of the aerosol droplets in the dorsal region (region 1) due to inertial impaction and gravity, while the ventral region (region 4) remains unexposed. To obtain a more homogenous distribution of aerosol droplets, the flow rate and droplet size should be considered together. Both affect droplet behavior and relevant forces. The impaction parameter is just one of the factors that may affect the droplet distribution pattern. When changing the impaction parameter, the deposition pattern of the droplets will change, but for a more realistic prediction, all effective parameters (e.g. droplet size, flow rate, viscosity, surface tension and temperature gradient) should be considered. Here, we change only the values of the impaction parameters. For larger droplets, the extent of deposition of droplets in the dorsal region increases, due to inertial impaction and gravitational force. In clinical practice, it was recently recommended to increase the flow rate from 0.5 mL s^−1^ to 0.6 mL s^−1^. On one hand, a higher flow rate of liquid will increase the initial velocity (assuming a constant droplet diameter), the inertial impaction and the deposition to the region opposite the nebulizer. On the other hand, increasing the flow rate from the nebulizer will lead to a smaller droplet size, which may improve spatial distribution of the aerosol. According to Fig. 10, the clinical device generates a mean droplet diameter range between 25 and 50 µm for different liquid flow rates. Results show that 0.6 mL s^−1^ can be an optimum flow rate of nebulization for the current PIPAC setup, considering the size of generated droplets. The homogeneity of distribution of aerosol droplets for 0.6 mL s^−1^ is slightly better than 0.5 mL s^−1^. Consequently, the recommendation for increasing the flow rate to 0.6 mL s^−1^ may help to obtain a more homogenous distribution of aerosol droplets during PIPAC, although the effect is rather modest.

**Table 1:** Measured mean diameters of the aerosol droplets for different flow rates.

Case number	*Q* (mL s^−1^)	*d*_*p*_ (µm)	d_p_^2^Q [µm^2^ mL s^−1^]
Case 1	0.4	48	921
Case 2	0.5	40	800
Case 3	0.6	35	735
Case 4	0.7	29	588
Case 5	0.8	28	627
Case 6	0.9	28	705

### Effect of liquid flow rate on nebulization cone angle

**Figure 12:**
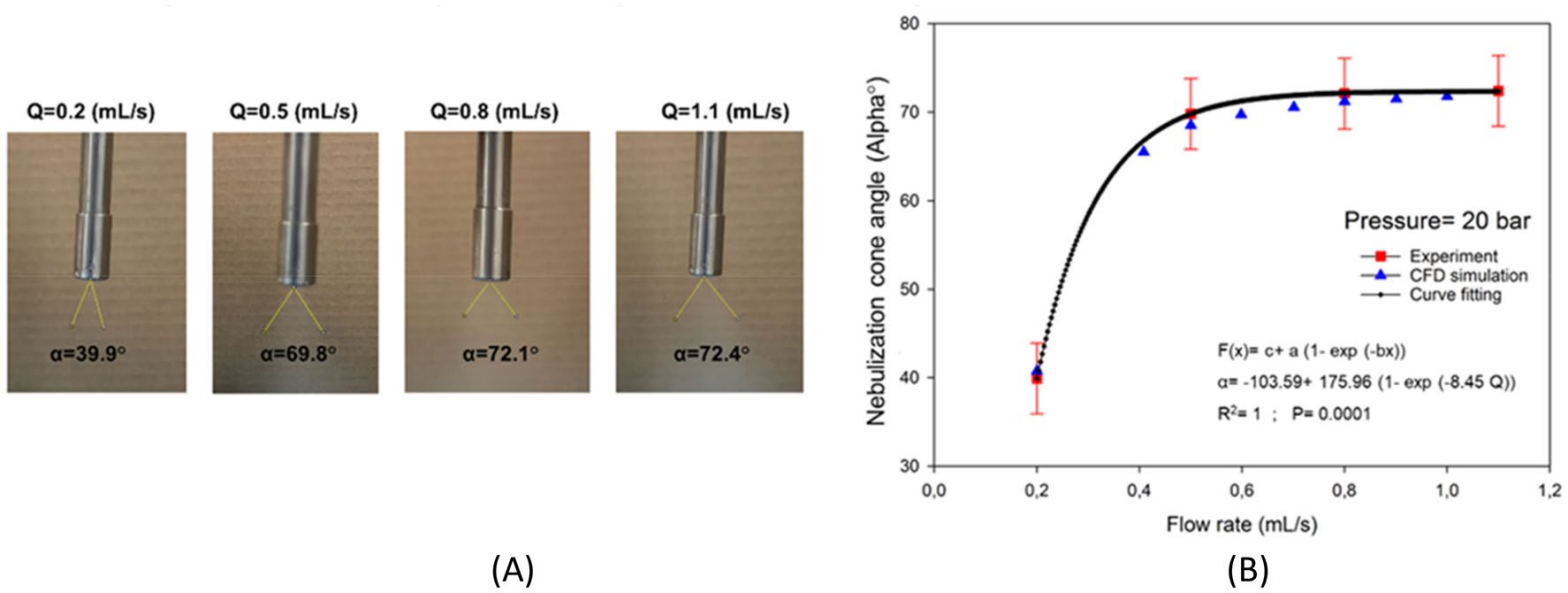
(**A**) The results of experiments to measure the cone angle of nebulization, and (**B**) A comparison of the results of experiments and CFD simulation for cone angle of nebulization and a curve fitting graph for trend of the results. (for the experiments, flow rates of 0.2, 0.5, 0.8 and 1.1 (mL s^−1^) and for the CFD simulations, flow rates of 0.2, 0.4, 0.5, 0.6, 0.7, 0.8, 0.9 mL s^−1^ and 1 mL s^−1^ were considered).

Figure 12A displays how aerosol droplets were nebulized through the nozzle experimentally and indicates the nebulization cone angle. This figure depicts the results of the experiments to measure the cone angle versus flow rate during in-vitro PIPAC experiments. A comparison of cone angles between the results of experiments and CFD simulations is shown by Fig. 12B. For the experiments, flow rates of 0.20, 0.50, 0.80 and 1.1 mL s^−1^ and for the CFD simulations, flow rates of 0.20, 0.40, 0.50, 0.6, 0.70, 0.80, 0.90 and 1 mL s^−1^ were considered. To measure the cone angle in the CFD model, a measure accumulator was defined in the COMSOL Multiphysics and two edges were drawn from the nozzle tip to the outer periphery of the spray. As shown, at first, the angle increases significantly with increasing flow rates until reaching its maximum value after which it remains almost constant. Indeed, the cone angle for current PIPAC strategies with a maximum pressure of 20 bar and a flow rate of 0.5 mL s^−1^ (or 0.6 mL s^−1^) is around 70°. As can be seen in this figure, there is an exponential relationship between cone angle and flow rate as well. Using the Curve Fitting Function in MATLAB® (R2018b, MathWorks, Natick, MA), an exponential function (*α* = − 103.59 + 175.96e^− 8.45^*Q*) was found for the relationship between the experimentally obtained cone angle of nebulization and flow rate.

### Effect of liquid viscosity

**Figure 13:**
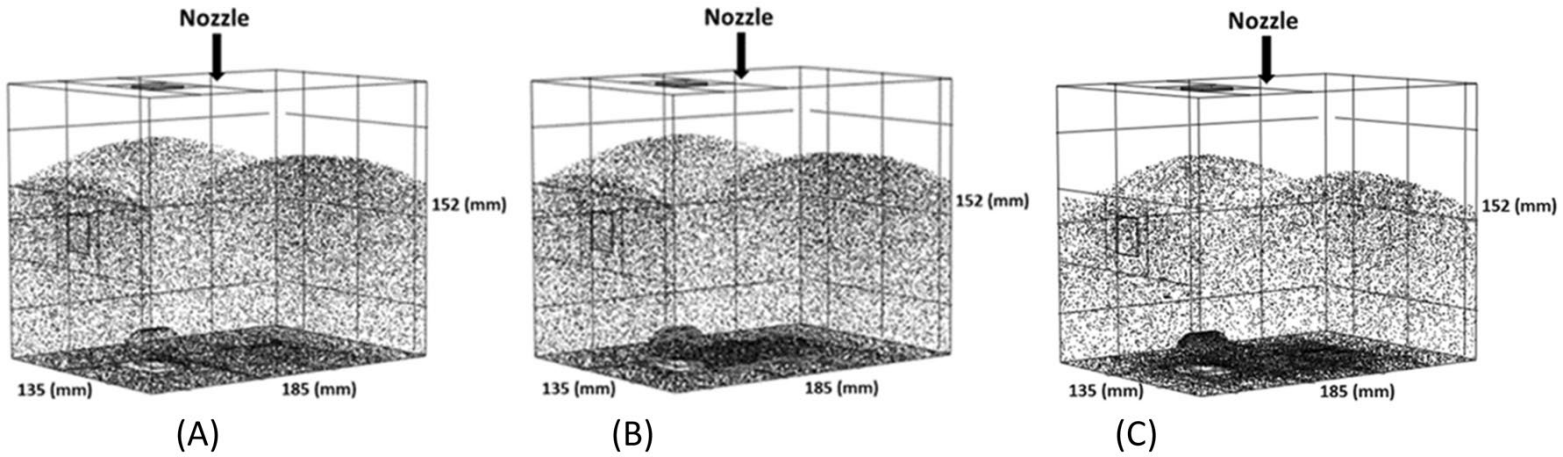
Visualization of aerosol droplet deposition with varying the viscosity of liquid the CFD box model, (**A**) Saline (NaCl 0.9%), (**B**) Icodextrin 4%, and (**C**) Icodextrin 7.5%. The black dots represent the deposited aerosol droplets. With increasing the viscosity of liquid, more aerosol droplets deposited at the bottom region of the box model (dorsal region).

**Figure 14:**
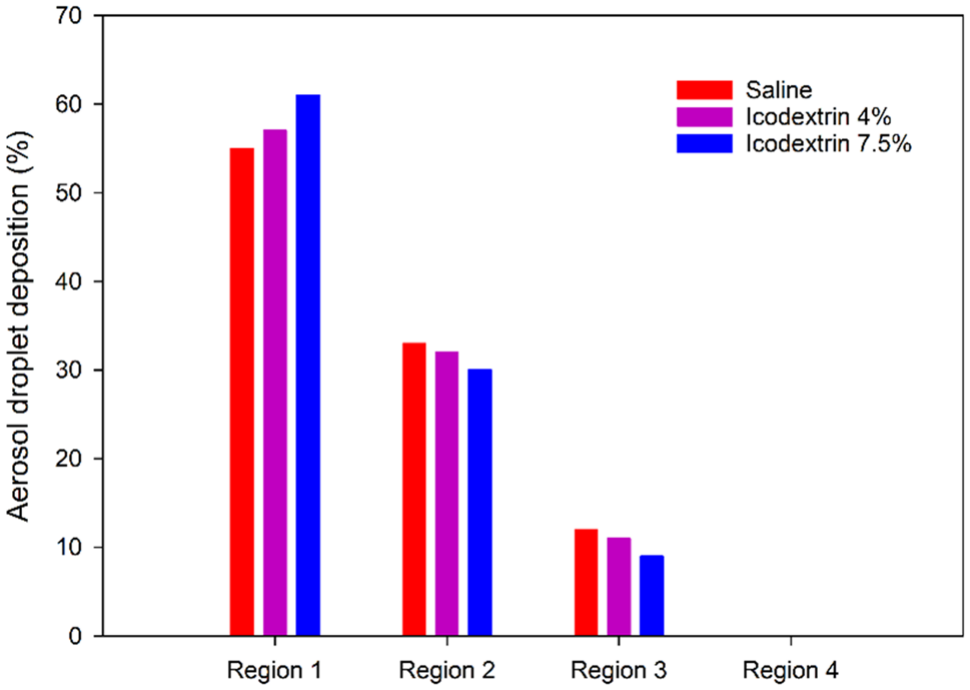
Nebulization of saline and Icodextrin 4% and 7.5% using CFD.

Dynamic viscosity measurements of Icodextrin resulted in values of 1.88 and 2.24 mPa s for concentrations of 4% and 7.5%, respectively. Keck et al.^35^ performed a granulometric analysis for nebulized Icodextrin using Capnopen®. Their results showed that the droplet size did not vary considerably with Icodextrin concentration: the median droplet diameter measured at two different Icodextrin concentrations (4% and 7.5%) was around 30 µm. Consequently, for the present CFD simulation, the droplet diameter was set at 30 µm with a flow rate of 0.5 mL s^−1^. Figures 13 and 14 show the results of CFD simulations of aerosol droplets being nebulized with different viscosities (saline (µ = 1 mPa s), Icodextrin 4% and 7.5%). As shown in these figures, at the highest viscosity (Icodextrin 7.5%), more aerosol droplets were deposited at the bottom (dorsal) region of the box model. The results of the CFD simulation for saline and Icodextrin solutions (4% and 7.5%) proved that the aerosol droplet distribution for liquid with lower viscosity is more homogenous than the liquid with higher viscosity.

### Effect of electrical potential (voltage) during Epipac

**Figure 15:**
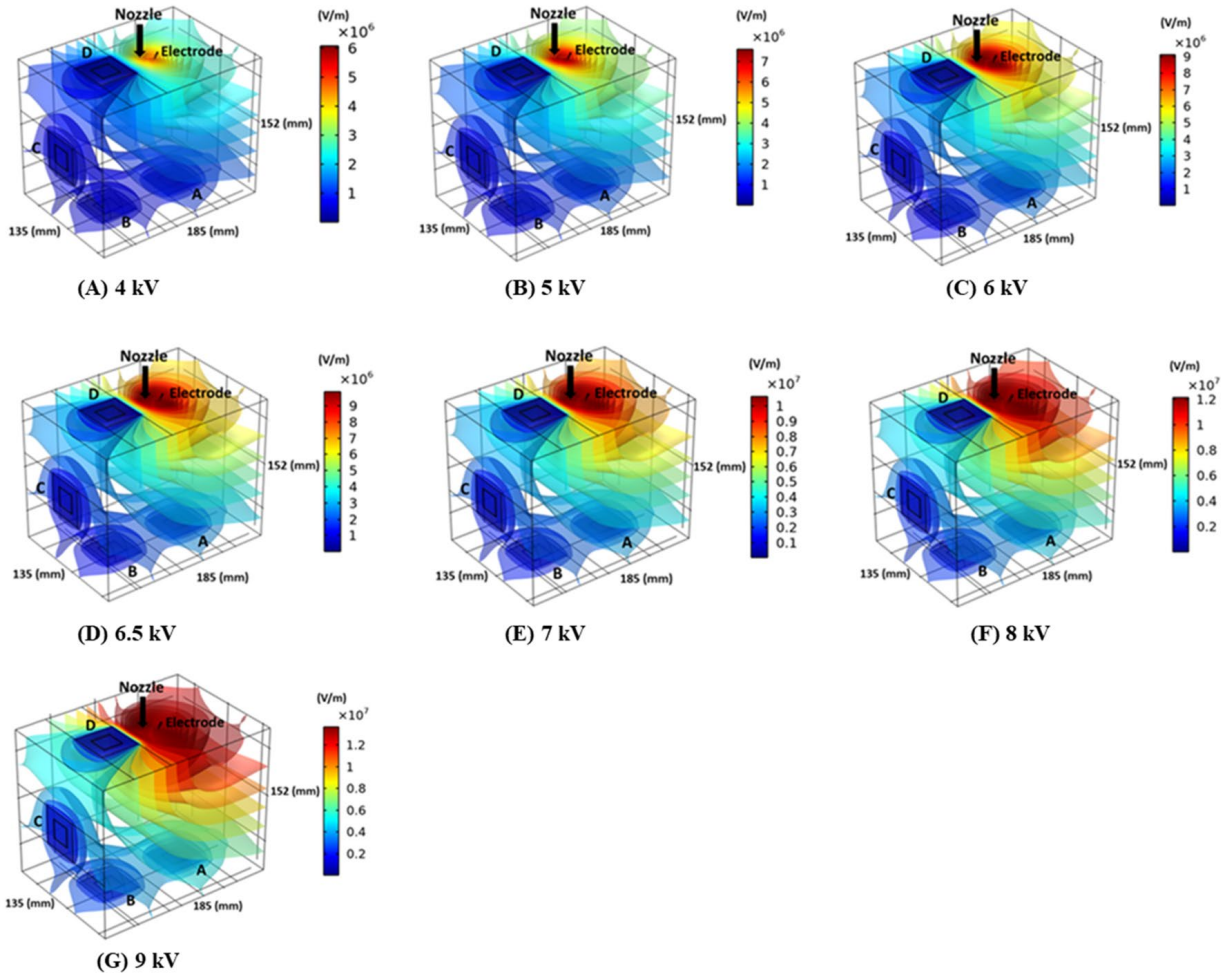
Visualization of the electrostatic field in the box model for (**A**) 4 kV, (**B**) 5 kV, (**C**) 6 kV, (**D**) 6.5 kV, (**E**) 7 kV, (**F**) 8 kV and (**G**) 9 kV.

**Figure 16:**
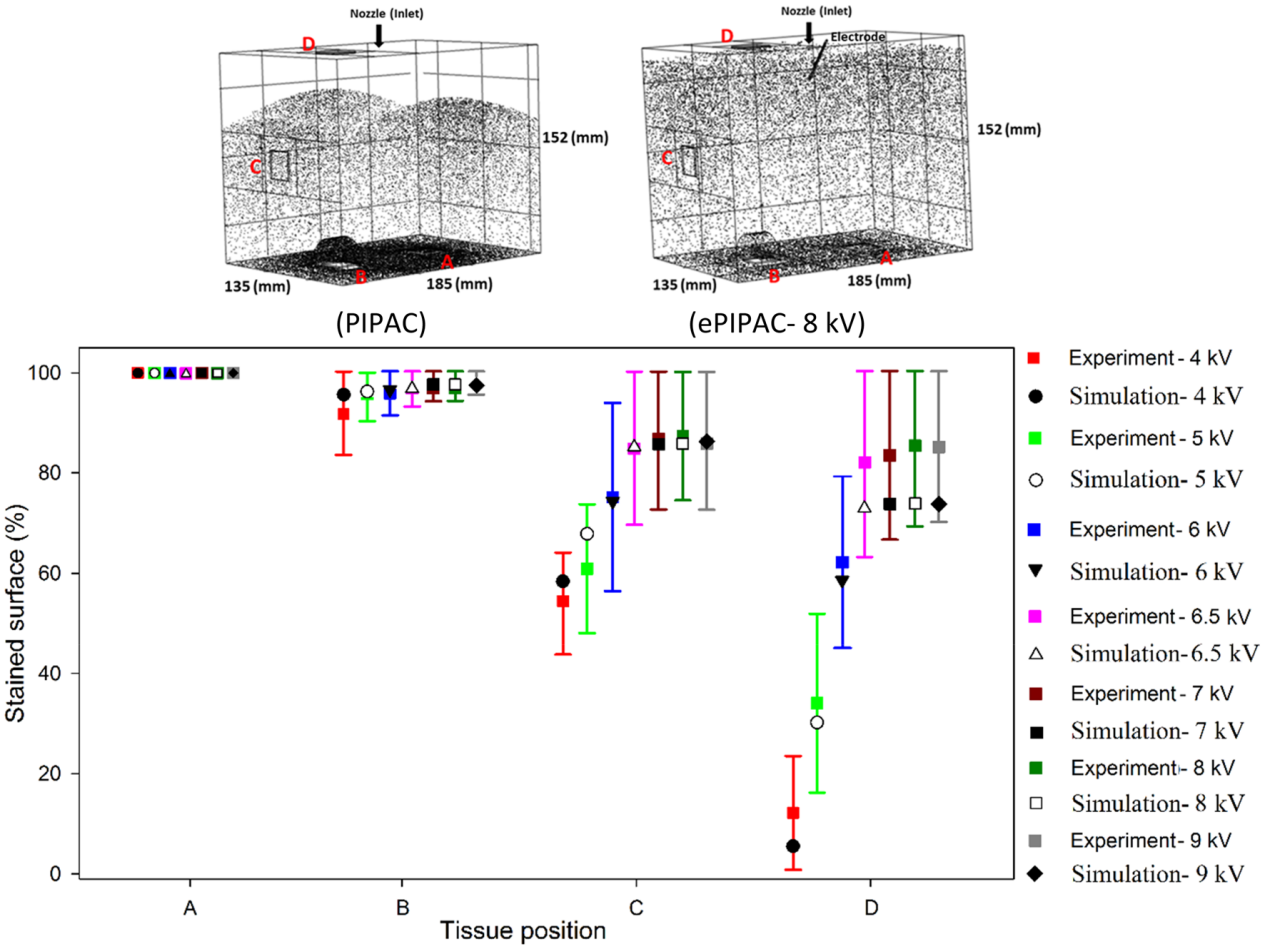
A comparison of the stained tissue surfaces (positions A–D) with black ink for different electrical potentials during ePIPAC between in vitro experiments and CFD simulations. Bars represent mean (± SD); experiments were repeated four times.

Figure 15 displays the electrostatic field in the box model at different electrical potentials (4, 5, 6, 6.5, 7, 8 and 9 kV). The electrical field (E = v/m) is the ratio of electrical potential (voltage) and distance (meter) and consequently, electrical force has a direct relationship to electrical potential. Increasing the electrical potential lead to an increase of the intensity of the electrostatic field in the box model. The charge of the droplets q were assumed to be − 1 for the simulation. It is obvious that the electrostatic force close to the brush electrode is higher than that further away. Figure 16 compares the in vitro experiments and CFD simulation results of the spatial distribution of black ink (the percentage of tissue surface ink staining for regions A-D) for different electrical potentials. Imposing an electrostatic field to the PIPAC setup (ePIPAC) results in a more homogenous aerosol droplet distribution in the box. A significant increase for proportion of black ink (a minimum of 60%) was observed at the stained tissue surface on the top wall of the box. The results showed an overall good agreement for the experiments and simulation. The electrical potential sensitivity analysis revealed that the aerosol droplet distribution became more homogenous as the electrical potential increased, but no further improvements were obtained after 6.5 kV. Consequently, the optimum electrical potential for ePIPAC seemed to be 6.5 kV, considering black ink as nebulized liquid. Both the CFD model and the experimental results show a significantly better aerosol deposition at the top of the box (plate D) when the electrical potential reached 6.5 kV. It is notable that this effect depended on the type of aerosol droplets and their electrical charge.

## Conclusions

Aerosolized intraperitoneal drug delivery holds considerable promise for the treatment of peritoneal cancer. The results of the current study contribute to our understanding of the effects of different parameters on aerosol droplet behavior in the peritoneal cavity, and may guide a rational choice of treatment parameters during clinical PIPAC procedures. We found that spatial distribution is optimal with small droplet sizes (1–5 µm). Using the current clinically used technology (droplet size of 30 µm), the optimal spatial distribution of aerosol is obtained with a flow rate of 0.6 mL s^−1^. The nebulization cone angle increases exponentially with flow rate, but a plateau is reached at 0.6 mL/s. Compared to saline, nebulization of higher viscosity liquids results in less homogeneous aerosol distribution. The addition of electrostatic precipitation significantly improves homogeneous aerosol distribution, but no further improvement is obtained with voltages higher than 6.5 kV. Further work will include the use of a realistic in vitro and CFD geometry, based on the actual anatomy, modelling of turbulence, and the use of high speed microscopic imaging to study the interaction of the aerosol droplets with the target tissues.
